# How an Antimicrobial Stewardship Team Treated a Nocardia farcinica-Associated Brain Abscess: A Case Report

**DOI:** 10.7759/cureus.54605

**Published:** 2024-02-21

**Authors:** Tetsushi Amano, Tomohide Nishikawa, Keisuke Oka, Kosei Ota, Taro Shimizu

**Affiliations:** 1 Antimicrobial Stewardship Team, Hekinan Municipal Hospital, Hekinan, JPN; 2 Department of Pharmacy, Hekinan Municipal Hospital, Hekinan, JPN; 3 Department of Neurosurgery, Hekinan Municipal Hospital, Hekinan, JPN; 4 Department of Infectious Diseases, Nagoya University Hospital, Nagoya, JPN; 5 Department of Clinical Laboratory, Hekinan Municipal Hospital, Hekinan, JPN; 6 Department of Diagnostic and Generalist Medicine, Dokkyo Medical University Hospital, Mibu, JPN

**Keywords:** microbiological technologist, diagnosis, pharmacist, nocardia farcinica, brain abscess, antimicrobial stewardship team

## Abstract

*Nocardia* species, which are ubiquitous in the environment, form lesions primarily in immunocompromised patients via oral or cutaneous infection. Some of these *Nocardia* species, such as* N.* *farcinica*, also infect the central nervous system via hematogenous dissemination, which rarely results in brain abscesses. Notably, *N.* *farcinica* is resistant to numerous antimicrobial drugs used in empirical therapy, necessitating the intervention of an infectious disease specialist. To date, no case of antimicrobial stewardship teams (ASTs) playing a central role in community hospitals without an infectious disease specialist has been reported. Here, we describe a case of *N. farcinica*-associated brain abscess in a small-to-medium-sized hospital with no infectious disease department or specialist, in which the AST assisted in the identification of the causative organism and in selecting appropriate therapeutic agents, ultimately leading to a cure.

The patient was an 88-year-old man with a high fever. He had been taking prednisolone (10-15 mg/day) for approximately 1 year for pemphigoid. Considering the possibility of fever owing to bacteremia of cutaneous origin, ampicillin/sulbactam antimicrobial therapy at 6 g/day was initiated. A subsequent close examination led to the diagnosis of a brain abscess. Emergency abscess drainage was performed by a neurosurgeon, and postoperative antimicrobial combination therapy comprising ceftriaxone (4 g/day), vancomycin (2 g/day), and metronidazole (1,500 mg/day) was commenced. The AST suspected *Nocardia* infection earlier, but further testing was difficult to perform at this facility. Therefore, by requesting assistance from Nagoya University Hospital, we performed early bacterial identification by mass spectrometry and appropriate antimicrobial susceptibility testing by a custom panel on day 11. The patient was non-responsive to all the previously used antibiotics at the time of admission. On day 13 after admission, the patient was successfully treated with trimethoprim-sulfamethoxazole (TMP-SMX) and imipenem/cilastatin sodium, and the patient was cured.

The AST can be as effective as an infectious disease specialist when a strong working relationship is established between the team and clinicians. Further, the activities of the AST can improve patient survival via active medical support in collaboration with attending physicians.

## Introduction

*Nocardia* is a common genus found in fresh and saltwater and the soil, caves, dust, decaying plants, and animal feces [1,2]. It has a characteristic beaded branching cell morphology and is gram-positive, aerobic, partially acid-fast, lysozyme-resistant, and catalase-positive. Currently, 109 *Nocardia* species, of which approximately half are considered clinically relevant, have been identified, and some of these species were initially isolated from human sources [2]. Further, *Nocardia* species infect humans via two main routes: respiratory tract infection through direct or indirect inhalation of cells and the skin following surgical procedures or traumatic skin injury. *Nocardia* species are also known to cause hematogenous dissemination from the lungs and skin to the brain, as well as pseudotumor lesions in the abdomen, eyes, and joints [3]. Infection of the central nervous system (CNS) due to hematogenous dissemination is observed in one-third of cases, while brain abscesses occur in approximately 9% of cases [4].

*Nocardia farcinica* is a highly invasive species with a strong tendency to infect the CNS and cause brain abscesses [5]. According to a systematic review, it accounts for 39.6% of all *Nocardia* cases [6]. It is also the most common strain in Japan and primarily infects individuals aged 60-80 years, with men reported to be three times more likely to be infected than women [7]. Further, *Nocardia* species can grow on blood agar and other media used in routine culture tests. However, its growth is slow and, in many cases, can be overlooked [1]. This makes their identification in general laboratories challenging. Therefore, 16S ribosomal RNA analysis and mass spectrometry using Matrix-Assisted Laser Desorption Ionization-Time of Flight Mass Spectrometry (MALDI-TOF MS) are often applied in this regard.

The activities of Antimicrobial Stewardship Teams (ASTs) include recommending appropriate specimen collection methods, selecting optimal antimicrobial agents based on the results of microbiological tests, and establishing the treatment administration period to ensure the prevention of recurrence. Thus, they play an important role in the rapid treatment of patients after diagnosis.

Here, we report a case of *N. farcinica*-associated brain abscess in a small-to-medium-sized hospital (255 beds) with no infectious disease department or specialist, in which the AST contributed to the early identification of the pathogenic microorganism and helped in the diagnosis, steps that ultimately led to the patient being cured.

## Case presentation

The patient was an 88-year-old man with a chief complaint being high fever. He had been taking prednisolone (10-15 mg/day) for approximately 1 year for pemphigoid, had a history of shingles and scabies, and had a pruritic rash on his trunk and lower extremities. He underwent surgery for lumbar spinal canal stenosis 12 years earlier. He had incontinence 10 days prior to admission and 6 days prior to admission, he was unable to stand up appropriately. Thus, he visited his local doctor for an examination.

**Figure 1:**
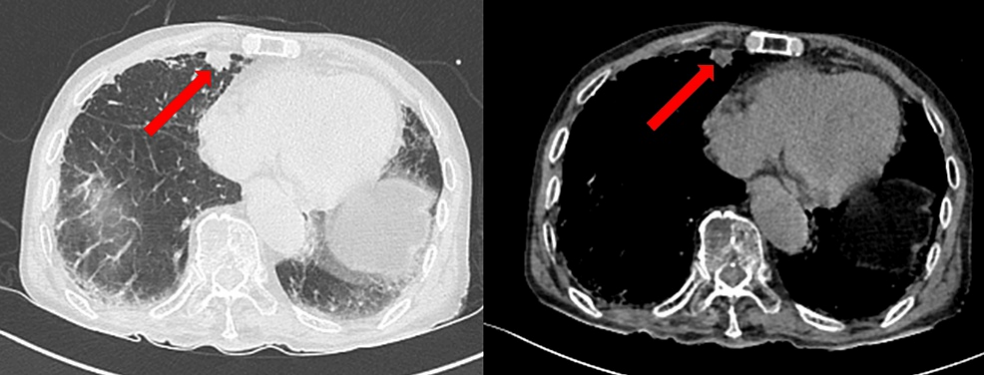
Chest CT showed a mass image on the mediastinal side of the lower middle lobe (red arrow) and no obvious pneumonia.

On admission, the vital signs of this patient were as follows: body temperature, 39.0 °C; blood pressure, 133/82 mmHg; sinus rhythm pulse, 100 beats/min; respiratory rate, 18 breaths/min; oxygen saturation, 89% (room air), and Glasgow Coma Scale, 13/15 (E4V4M5). Further, his white blood cell count, neutrophil rate, and C-reactive protein (CRP) concentration were 13,100/µL (reference range: 3,300-8,600/µL), 83.5%, and 8.58 mg/dL (reference range: <0.14 mg/dL), respectively. The patient showed no symptoms of headache or vomiting, and at the time of admission, he was disorientated but was able to respond when spoken to. His chest and abdomen computed tomography (CT) images showed emphysema and a mass image on the mediastinal side of the lower middle lobe; however, there was no obvious pneumonia or active inflammation of his abdomen (Figure 1).

**Figure 2:**
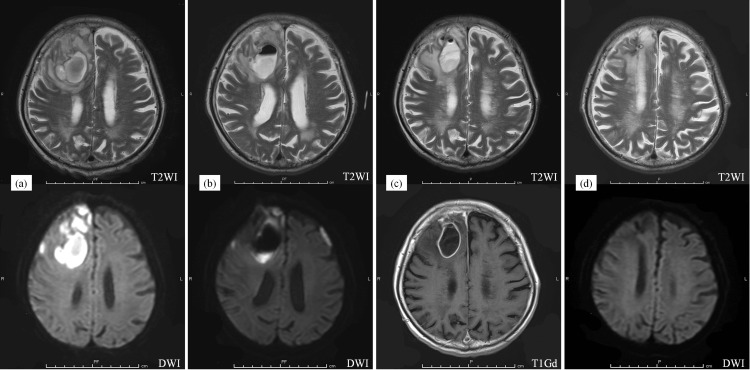
MRI scans obtained on different days after admission. (a) Day 4: a polycystic lesion in the right frontal lobe of the patient’s brain with high intensity on the T2-weighted image (T2WI) and diffusion-WI. (b) Day 11: shrinkage of the brain lesion. (c) Day 39: fluid re-accumulation in the lesion space after drainage. On the right side is a gadolinium-enhanced T1 weighted-image (T1Gd). (d) Day 193: shrinkage of lesion and non-recurrence of the abscess.

Since the source of the fever could not be determined on admission, ampicillin/sulbactam therapy (6 g/day) was initiated after considering the possibility of bacteremia of cutaneous origin owing to the numerous rashes and scratches observed on his skin. Further, given that the patient was hospitalized on the weekend, his head CT scan was performed the following week, on the fourth day of hospitalization. Notably, two space-occupying lesions with edema were observed in his right frontal lobe. Brain magnetic resonance imaging (MRI) further showed a hyperintense polycystic lesion on T2- and diffusion-weighted images (Figure 2a).

The patient was referred to the neurosurgery department, where emergency abscess drainage was performed. Thereafter, 15 mL of the cream-colored abscess was cultured, while postoperative antimicrobial combination treatment, comprising ceftriaxone (CTRX; 4 g/day), vancomycin (VCM; 2 g/day), and metronidazole (MNZ; 1,500 mg/day), was commenced.

**Figure 3:**
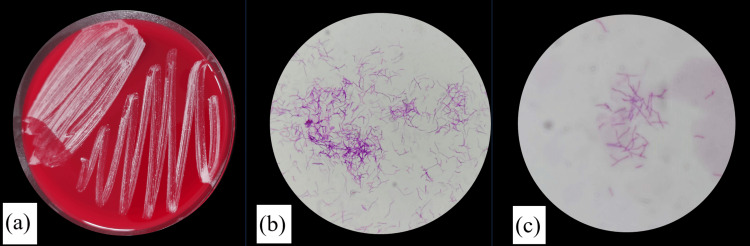
(a) Colonies on Sheep Blood Agar, (b) Gram-stained (× 1,000), and (c) Kinyoun-stained (× 1,000) image of brain abscess.

The AST microbiological technologist performed Gram staining on the bacilli on day 5 after admission and observed branched gram-positive rods. Further, Kinyoun staining showed weak anti-acidity, indicating the possibility of *Nocardia* (Figure 3). However, identifying the specific *Nocardia* species at our facility was challenging. Thus, these samples were sent to Nagoya University Hospital for further analysis.

The AST pharmacist took steps to quickly purchase trimethoprim-sulfamethoxazole (TMP-SMX) and imipenem/cilastatin sodium (IPM/CS), two intravenous antimicrobial agents, which were not in stock at our hospital given that they are not generally used at our hospital. Screening for human immunodeficiency virus infections recommended by the AST pharmacist was negative.

At Nagoya University Hospital, the bacteria were identified as *N. farcinica* via mass spectrometry (VITEK® MS; BIOMÉRIEUX, Tokyo, Japan) performed on day 8 after admission (99.8 confidence level). Based on the criteria set forth by the Clinical and Laboratory Standards Institute (CLSI), drug susceptibility testing performed on day 11 after admission revealed that the patient was resistant to all three drugs used for postoperative infection prevention (Table 1). Therefore, we decided to replace the combination therapy with TMP (720 mg/day) and SMX (3,600 mg/day) plus IPM/CS (1.5 g/day) on day 13 after admission. Before the change in the antimicrobial agents, the patient was referred to the Respiratory Medicine department for bronchoscopic alveolar lavage fluid examination in detail of the mass lesions in the lungs; *N. farcinica* was not detected in the collected lavage fluid or sputum. Two sets of blood cultures taken on admission were negative, and although the culture period was extended to three weeks, they were ultimately negative.

**Table 1:** Antimicrobial susceptibility of N. farcinica (Clinical and Laboratory Standards Institute susceptibility standard M62-1st). MIC: minimum inhibitory concentration; S: susceptible; R: resistant; I: intermediate

Antimicrobial agent	MIC (μg/mL)	Interpretation
Amoxicillin-clavulanate	4	S
Ceftriaxone	>32	R
Imipenem	1	S
Amikacin	≤8	S
Clarithromycin	>16	R
Doxycycline	>1	I or R
Minocycline	≤1	S
Ciprofloxacin	≤1	S
Moxifloxacin	≤1	S
Sulfamethoxazole-trimethoprim	2	S

The patient was monitored via blood tests and imaging evaluations regularly, and an MRI on day 39 after admission showed fluid accumulation in his brain, where the first drainage was performed (Figure 2b, 2c). Further, the patient had a mild fever (37.7 °C). Even though the fever was previously resolved, the CRP increased from 0.19 to 4.09 mg/dL. On day 42, after admission, a second abscess drainage was performed, and 7 mL of cream-colored purulent fluid was collected and examined. Analysis in this regard showed that the drained pus cultures did not contain microorganisms, including *N. farcinica*. The patient showed good progress after this second surgery, and on day 57 after admission, he was placed on an oral TMP-SMX (320 mg/1,600 mg) monotherapy twice daily. In accordance with a previous report that the duration of antimicrobial therapy should be 4-8 weeks for intravenous administration and at least 6 months for long-term oral administration [5,8], we administered the drug intravenously for 6 weeks before reducing the dose by two-thirds for oral administration. At 111 days after admission, he was discharged from the hospital and was continued on oral medications. He received outpatient medical therapy and follow-up imaging, and an MRI performed on day 193 after admission showed that the lesion had shrunk and had not recurred (Figure 2d). The duration of treatment was initially planned to be 12 months, but since prednisolone was still being administered, the duration of administration was further extended. The administration was terminated after an MRI on day 473 confirmed the disappearance of the abscess. 

## Discussion

In this report, we present a case of a brain abscess owing to an *N. farcinica* infection identified by a well-functioning AST at a small-to-medium-sized hospital established in 2017. To date, no reports have been published on AST interventions with respect to diagnostic and therapeutic support. Only a few cases of brain abscesses are present at our hospital each year, and this was the first case of a *Nocardia*-associated brain abscess in 35 years since the hospital was opened.

Brain abscesses caused by *Nocardia* species, which account for only 2% of all intracranial abscesses [9,10], are frequently overlooked during initial diagnosis. Further, *N. farcinica* infection is lethal in 55% of immunocompromised and 20% of immunocompetent patients and has a poor prognosis in more than 30% of patients who receive inadequate empirical antimicrobial therapy [3,11]. Furthermore, *N. farcinica* can be isolated from clinical specimens, such as pus, sputum, bronchial secretions, and skin [12]. However, the identification of *Nocardia* species may be challenging in some cases, given that they grow slowly and require weeks to form colonies [2].

For our patient, smear findings provided a very important clue that allowed the AST microbiological technologist to make an early assumption of *Nocardia*. In general, identifying bacterial species is often challenging for hospitals that are not well equipped. Further, given that a considerable amount of time is required for analysis and that testing at a specialized institution is often necessary, we requested an examination at the Nagoya University Hospital. Further, the AST pharmacists demonstrated their true worth by obtaining and selecting the necessary antimicrobial agents and establishing dose regimes. With the exception of amikacin (AMK), *N. farcinica* is resistant to ampicillin, broad-spectrum cephalosporins, clarithromycin, and aminoglycosides. In general, bacteria-induced brain abscesses are treated empirically using third-generation cephalosporins, MNZ, and VCM before susceptibility test results are available [13]. *Nocardia* species are typically resistant to these agents. Thus, triple therapy using TMP-SMX, AMK, and CTRX or IPM/CS is recommended [1,14]. CTRX monotherapy is rarely effective against *N. farcinica* [15,16]. Many small and medium-sized hospitals have access to only one agent of carbapenems; at our hospital, the AST could use only meropenem, which shows activity against *N. farcinica* [1]. However, for the treatment of this patient, we considered another carbapenem, IPM/CS, which had sufficient evidence in the literature but had not been previously used at our hospital [8]. Intravenous infusion of TMP-SMX had also not been previously adopted; therefore, we adopted it as we did IPM/CS. TMP-SMX is the first-line drug in standard therapy for *N. farcinica*, given that it crosses the blood-brain barrier, shows excellent tissue migration ability, and has been used in several cases in the past. Even though there has been some debate regarding *N. farcinica* showing TMP-SMX resistance, no substantial evidence exists in this regard [2].

When *N. farcinica* was identified, the antimicrobial therapy was not changed. Rather, we prioritized the identification of pulmonary lesions, and after obtaining drug susceptibility results, we optimized the therapy using a combination of TMP-SMX and IPM/CS. The creatinine clearance in this case was > 50 mL/min, and AMK could be used while adjusting the dose with therapeutic drug monitoring. However, since this patient was very old, we excluded the use of AMK from the combination therapy in consideration of worsening renal function. Tetracycline is the drug of choice when TMP-SMX cannot be used owing to allergy. However, tetracycline was not selected in this case because the patient had no history of allergy. It is also worth emphasizing that doxycycline was not used as it is only effective against *N. farcinica* in 2% of patients [17]. Further, moxifloxacin, a fluoroquinolone, and linezolid, an oxazolidinone, are effective in vitro [18]; however, we retained them as second-line treatments owing to limited clinical experience with their use and the fact that the use of antimicrobial agents generally requires a lot of cautions. Even though the optimal treatment was delayed for approximately 3 days, the prognosis of the patient basically remained unaffected, given that the disease progressed at a slower rate than is generally observed for a bacterial infection-related brain abscess.

The activities of the AST, particularly those of the microbiological technologist and pharmacist, as well as the rapid surgical treatment after the diagnosis of the brain abscess, were crucial for the successful treatment of our patient. The AST, which can be involved in antimicrobial and diagnostic stewardship, is indispensable for community hospitals without infectious disease specialists [3]. Although the structural problem of insufficient staff is a disadvantage for AST activities in small and medium-sized hospitals, it is possible to fully take advantage of close proximity and easy communication among staff. AST pharmacists trained in infectious disease practice will be able to propose appropriate antibiotic dosing designs to many physicians, making full use of pharmacokinetics and pharmacodynamics parameters and therapeutic drug monitoring. By continuing to consult with physicians on a daily basis on all aspects of infectious disease care, and by treating them sincerely, you will earn the trust of physicians. Their activities in the current case included the early detection and identification of pathogenic microorganisms, recommendation of appropriate specimen collection, selection of the optimal antimicrobial agents based on the results of microbiological tests, and setting the treatment administration period to prevent recurrence.

For nocardiosis, the median time from admission to is 42 days (range, 9-120 days), with patients with underlying disease showing particularly longer treatment durations (up to 53.8 days) [19]. Although this report is limited to *N. farcinica*-associated brain abscesses, it can be said that the activities of the AST allowed for very early diagnosis compared to the time taken for diagnosis of brain abscesses reported in previous studies [5,19]​​​​​​.

## Conclusions

In this study, we report the case of an 88-year-old man who presented with high fever as the primary complaint and was diagnosed with an *N. farcinica* infection. This patient showed no response to various antimicrobial regiments, but owing to the activities of the AST, the *Nocardia* infection was diagnosed early, on day 13 of admission, and an optimal treatment dose and duration were proposed. Therefore, the AST can be as beneficial as an infectious disease specialist, and their activities, in collaboration with attending physicians, can lead to improved patient survival. Furthermore, we believe that the fusion of antimicrobial and diagnostic stewardship activities can contribute to improving the long-term survival rate.

## References

[1] Brown-Elliott BA, Brown JM, Conville PS, Wallace RJ Jr (2006). Clinical and laboratory features of the Nocardia spp. based on current molecular taxonomy. Clin Microbiol Rev.

[2] Traxler RM, Bell ME, Lasker B, Headd B, Shieh WJ, McQuiston JR (2022). Updated review on Nocardia species: 2006-2021. Clin Microbiol Rev.

[3] Kumar VA, Augustine D, Panikar D, Nandakumar A, Dinesh KR, Karim S, Philip R (2014). Nocardia farcinica brain abscess: epidemiology, pathophysiology, and literature review. Surg Infect (Larchmt).

[4] Sánchez-Ramos D, Vega-Torres E, Irles-Vidal C, Colomina-Rodríguez J (2022). Cerebellious abscesses caused by Nocardia farcinica in an immunocompromised patient. Rev Esp Quimioter.

[5] Beucler N, Farah K, Choucha A, Meyer M, Fuentes S, Seng P, Dufour H (2022). Nocardia farcinica cerebral abscess: a systematic review of treatment strategies. Neurochirurgie.

[6] Meena DS, Kumar D, Bohra GK, Midha N, Garg MK (2022). Clinical characteristics and treatment outcome of central nervous system nocardiosis: a systematic review of reported cases. Med Princ Pract.

[7] Kageyama A, Yazawa K, Ishikawa J, Hotta K, Nishimura K, Mikami Y (2004). Nocardial infections in Japan from 1992 to 2001, including the first report of infection by Nocardia transvalensis. Eur J Epidemiol.

[8] Margalit I, Lebeaux D, Tishler O, Goldberg E, Bishara J, Yahav D, Coussement J (2021). How do I manage nocardiosis?. Clin Microbiol Infect.

[9] Grond SE, Schaller A, Kalinowski A, Tyler KA, Jha P (2020). Nocardia farcinica brain abscess in an immunocompetent host with pulmonary alveolar proteinosis: a case report and review of the literature. Cureus.

[10] Fatahi-Bafghi M (2018). Nocardiosis from 1888 to 2017. Microb Pathog.

[11] Corti ME, Villafañe-Fioti MF (2003). Nocardiosis: a review. Int J Infect Dis.

[12] Budzik JM, Hosseini M, Mackinnon AC Jr, Taxy JB (2012). Disseminated Nocardia farcinica: literature review and fatal outcome in an immunocompetent patient. Surg Infect (Larchmt).

[13] Alvis Miranda H, Castellar-Leones SM, Elzain MA, Moscote-Salazar LR (2013). Brain abscess: current management. J Neurosci Rural Pract.

[14] Lerner PI (1996). Nocardiosis. Clin Infect Dis.

[15] Ukai Y, Fujimoto N, Fujii N (2012). Case of muscle abscess due to disseminated nocardiosis in a patient with autoimmune hemolytic anemia, and review of the published work. J Dermatol.

[16] Wallace RJ Jr, Tsukamura M, Brown BA, Brown J, Steingrube VA, Zhang YS, Nash DR (1990). Cefotaxime-resistant Nocardia asteroides strains are isolates of the controversial species Nocardia farcinica. J Clin Microbiol.

[17] Cortés P, Jane Hata D, Libertin C, Meza Villegas DM, Harris DM (2021). Cladophialophora bantiana and Nocardia farcinica infection simultaneously occurring in a kidney transplant recipient: case report and literature review. Immun Inflamm Dis.

[18] Moylett EH, Pacheco SE, Brown-Elliott BA (2003). Clinical experience with linezolid for the treatment of nocardia infection. Clin Infect Dis.

[19] Yang M, Xu M, Wei W (2014). Clinical findings of 40 patients with nocardiosis: a retrospective analysis in a tertiary hospital. Exp Ther Med.

